# Sufentanil increases delirium risk compared to fentanyl in elderly perioperative ischemic stroke patients: a retrospective cohort study

**DOI:** 10.3389/fmed.2026.1770366

**Published:** 2026-02-12

**Authors:** Yanan He, Mengyao Qu, Yuxiang Song, Rui Wang, Peng Li, Yingfu Li, Yixun Lu, Miao Sun, Huikai Yang, Hang Guo, Weidong Mi, Yulong Ma

**Affiliations:** 1 Department of Anesthesiology, The First Medical Center of Chinese PLA General Hospital, Beijing, China; 2 Nation Clinical Research Center for Geriatric Diseases, Chinese PLA General Hospital, Beijing, China; 3 Department of Anesthesiology, The Sixth Medical Center of Chinese PLA General Hospital, Beijing, China; 4 Department of Anesthesiology, The Seventh Medical Center of Chinese PLA General Hospital, Beijing, China; 5 The Second School of Clinical Medicine, Southern Medical University, Guangzhou, China

**Keywords:** delirium, fentanyl, opioid analgesics, stroke, sufentanil

## Abstract

**Background:**

Perioperative ischemic stroke (PIS) and postoperative delirium (POD) are each serious central nervous system complications. When they occur together, they present a potentially devastating but largely understudied condition. Evidence on risk factors for this combined pathology is scarce. This study examined the incidence and prognosis of PIS complicated by POD and evaluated whether intraoperative opioid choice, fentanyl versus sufentanil, affects the risk of this dual complication.

**Methods:**

We retrospectively analyzed 376,933 patients who underwent non-cardiac surgery between January 2008 and August 2019. After applying exclusion criteria, 525 patients with PIS were identified. Of these, 178 elderly patients (≥ 65 years) formed the study cohort. Kaplan–Meier survival curves were used to compare overall survival between patients with and without POD, with group differences assessed by the log-rank test. Patients were categorized into a fentanyl group (*n* = 73) and a sufentanil group (*n* = 105). The primary outcome was POD within 7 days after surgery. Multivariate logistic regression examined the association between opioid type and POD risk, adjusting for patient- and surgery-related confounders. Additionally, 1:1 propensity score matching (44 matched pairs) was used to balance baseline characteristics and confirm findings.

**Results:**

The overall POD incidence among elderly PIS patients was 40.4% (72/178). Patients who developed POD had obviously worse survival compared to those who did not (30% *vs.* 50%). The incidence of POD was markedly lower in the fentanyl group than in the sufentanil group (6.8% *vs*. 63.8%). Fully adjusted models showed fentanyl use was associated with a substantially reduced risk of POD (OR: 0.017, 95% CI: 0.003–0.070, *p* < 0.001). The lower POD risk with fentanyl compared to sufentanil remained consistent across subgroups stratified by age, sex, and diabetes status.

**Conclusion:**

In elderly patients with PIS, intraoperative fentanyl use was associated with a significantly lower risk of POD compared with sufentanil. Opioid selection may represent a modifiable risk factor in this high-risk population, offering an opportunity to improve anesthetic management and postoperative outcomes.

## Introduction

Postoperative delirium (POD) and perioperative ischemic stroke (PIS) are two major central nervous system complications in surgical patients, each carrying high morbidity and mortality. POD, defined by acute fluctuations in attention and awareness, affects 10–45% of general surgical patients, with even higher rates in vulnerable populations (1). PIS, on the other hand, markedly increases the risk of adverse outcomes such as prolonged hospitalization and death (2). Despite their clinical importance, the combined occurrence of PIS complicated by POD remains poorly studied, representing a substantial gap in perioperative neuroscience research.

The epidemiology, risk factors, and outcomes of delirium specifically in PIS patients are not well-defined. However, this group may experience compounded neurological insults, simultaneous cerebrovascular injury, and acute cognitive dysfunction (3). When present together, these conditions may have synergistically detrimental effects (4), leading to more severe cognitive decline and higher mortality than either complication alone. Identifying modifiable delirium risk factors in stroke patients is therefore a pressing clinical priority.

Recent work has expanded knowledge of PIS risk factors and predictive tools. Our research group established one of the largest multi-center perioperative stroke databases in China, analyzing over 220,000 non-cardiac surgery patients and identifying independent predictors such as coronary heart disease, elevated BMI, preoperative hyperglycemia, and specific biomarkers (5–11). However, the impact of anesthetic management—particularly intraoperative opioid choice—on delirium risk in PIS patients has not been examined. The association between ischemic stroke and delirium has been extensively documented in the general stroke population. Patients with acute ischemic stroke demonstrate markedly elevated susceptibility to delirium, with reported incidence rates ranging from 13 to 48% depending on stroke severity, location, and patient characteristics (12). This heightened vulnerability stems from shared pathophysiological mechanisms including cerebral hypoperfusion, disruption of blood–brain barrier integrity, neuroinflammatory cascades, neurotransmitter dysregulation, and oxidative stress (13). These mechanisms create a fragile neurological substrate particularly prone to cognitive perturbations. However, the specific relationship between perioperative ischemic stroke (PIS) and postoperative delirium (POD) remains inadequately characterized. In PIS patients, the temporal relationship between cerebrovascular injury and delirium onset may be complex and bidirectional, as surgical trauma, anesthetic exposure, and hemodynamic instability can both precipitate stroke and independently trigger delirium through overlapping pathophysiological pathways (14). This complexity underscores the critical importance of identifying modifiable perioperative factors - particularly anesthetic management strategies - that could reduce delirium burden in this exceptionally vulnerable population.

Opioid selection is a potentially modifiable factor in this high-risk population. While opioids are essential perioperative analgesia, emerging evidence suggests that specific agents may influence delirium risk through differences in pharmacokinetics, pharmacodynamics, cerebral blood flow regulation, and neuroinflammatory effects (15). Fentanyl and sufentanil, two commonly used opioids, have distinct properties that could differentially affect patients with impaired cerebral autoregulation following stroke (16).

The primary objective of this retrospective cohort study was to (i) describe the epidemiology of POD in patients with PIS, (ii) assess its impact on survival outcomes, and (iii) evaluate the association between intraoperative opioid choice (fentanyl vs. sufentanil) and POD risk in elderly PIS patients. By identifying modifiable perioperative factors, this study aims to inform evidence-based anesthetic strategies for improving outcomes in this vulnerable group.

## Materials and methods

### Study design and participants

**Figure 1:**
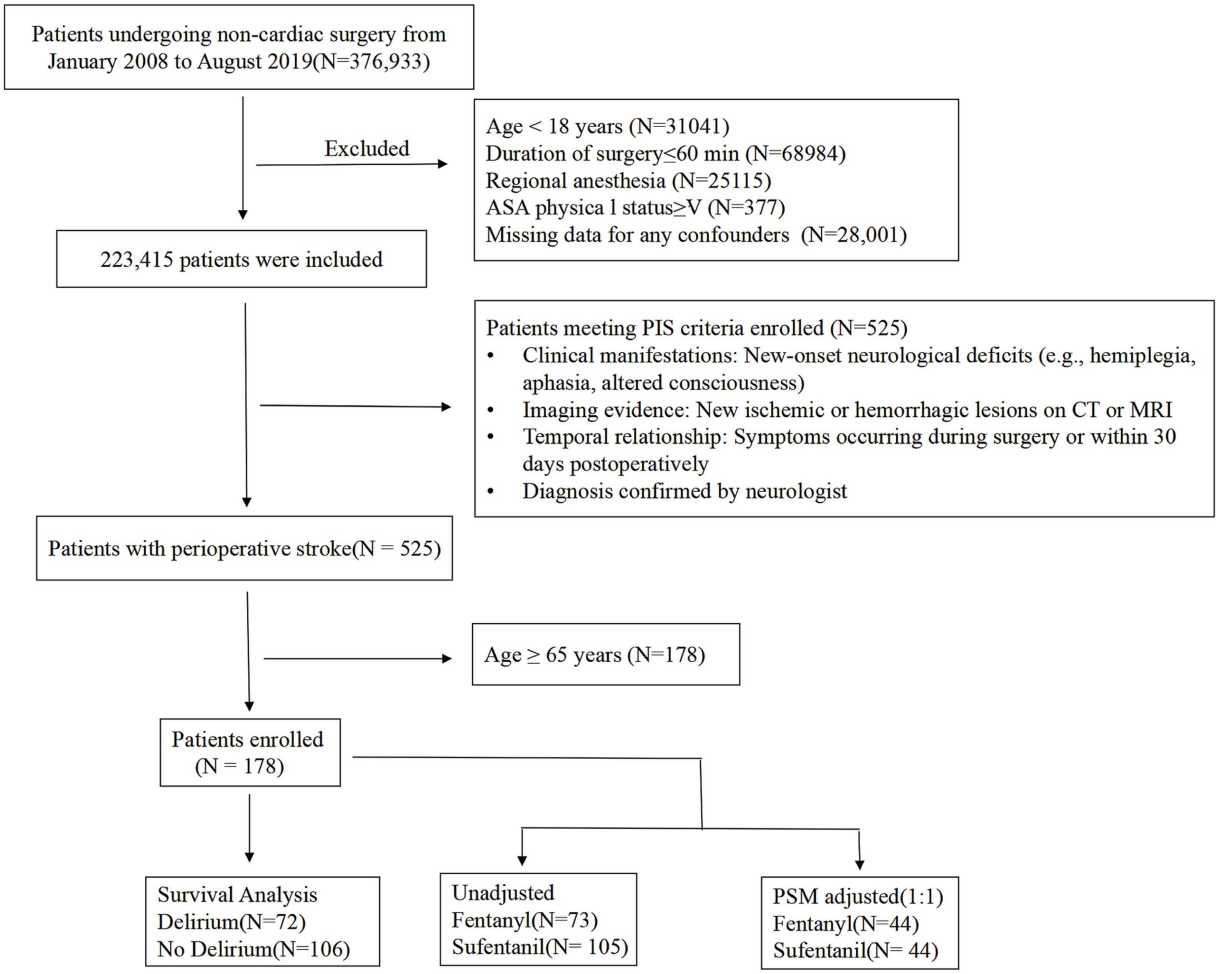
Study flow diagram. The diagram illustrates the systematic screening process from 376,933 non-cardiac surgery patients to the final cohort of 178 elderly patients with perioperative ischemic stroke (PIS). After propensity score matching (PSM), 88 patients (44 in each group) were included in the matched analysis. PIS, perioperative ischemic stroke; PSM, propensity score matching; POD, postoperative delirium.

This retrospective cohort study analyzed data from an extensive surgical database containing 376,933 patients who underwent non-cardiac surgery between January 2008 and August 2019. After applying systematic exclusions, 223,415 patients remained eligible for screening. Perioperative ischemic stroke (PIS) was identified in 525 patients based on clinical presentation, imaging findings, temporal association with surgery, and neurologist confirmation. Patients younger than 65 years were excluded, leaving 178 elderly patients with PIS for the final analysis. The screening process is illustrated in Figure 1.

The study was approved by the Ethics Committee of the Chinese PLA General Hospital (S2025-123-01) with a waiver of informed consent and was conducted by STROBE guidelines.

### Outcome and exposure measures

The primary exposure was the type of intraoperative opioid, fentanyl or sufentanil, administered during general anesthesia. Opioid selection was determined by attending anesthesiologist preference and drug availability, without a standardized institutional protocol. The primary outcome was postoperative delirium (POD) within 7 days of surgery.

PIS was defined as any neurological impairment affecting movement, sensation, or cognition caused by a localized blockage of blood supply in the brain, spinal cord, or retina within 30 days post-surgery (17). Stroke diagnoses were confirmed by at least one ICD-9-CM or ICD-10-CM code (Supplementary Table S1) documented in discharge records.

Secondary outcomes included overall survival, measured from the date of surgery to the date of death from any cause during the follow-up period.

### Definition of POD

POD was diagnosed by a neurologist based on descriptors such as altered mental status, confusion, disorientation, agitation, inappropriate behavior, inattention, hallucinations, and combative behavior (18). Diagnoses were supported by documentation in the anesthesiology department’s database, as described previously (18)^.^

Medical records were also reviewed for administration of quetiapine, olanzapine, haloperidol, or risperidone postoperatively. The diagnostic criteria followed the DSM-IV guidelines (19). Patients who exhibited delirium symptoms or received these medications in their preoperative records were excluded.

### Data collection

Preoperative variables included age, sex, and body mass index (BMI), and comorbidities such as hypertension, diabetes, coronary heart disease, previous ischemic stroke, atrial fibrillation, peripheral vascular disease, and renal dysfunction.

Surgical variables included American Society of Anesthesiologists (ASA) classification, intraoperative opioid type and dose, morphine and remifentanil dosage, estimated blood loss, transfusion requirements, and intraoperative fluid administration (colloid and crystalloid volumes). Use of dexmedetomidine and non-steroidal anti-inflammatory drugs (NSAIDs) during the perioperative period was also recorded.

Laboratory data included hemoglobin, total bilirubin, and glucose levels from the most recent test conducted within 3 days before surgery. Inflammatory markers, including neutrophil-to-lymphocyte ratio (NLR) and platelet-to-lymphocyte ratio (PLR), were calculated from complete blood counts. BMI was computed from measured height and weight.

POD assessments were performed by trained healthcare professionals using standardized screening tools for the first five postoperative days. Length of hospital stay was calculated from the date of surgery to discharge.

### Statistical analysis

Continuous variables with normal distribution were expressed as mean ± standard deviation (SD) and compared using Student’s t-test. Non-normally distributed variables were reported as median ± interquartile range (IQR) and compared using the Mann–Whitney U test. Categorical variables were presented as counts (percentages) and compared using a chi-squared test or Fisher’s exact test, as appropriate.

Kaplan–Meier survival analysis with log-rank testing was used to compare overall survival between patients with and without POD.

The association between intraoperative opioid type and POD risk was evaluated using univariate and multivariate logistic regression models. Opioid type (fentanyl vs. sufentanil) was treated as a binary exposure variable. Covariates were selected based on clinical relevance and prior literature identifying established risk factors for POD. Model 2 (patient-related confounders) included: sex, age, BMI, ASA physical status, hypertension, diabetes, coronary heart disease, previous ischemic stroke, atrial fibrillation, peripheral vascular diseases, renal dysfunction, hemoglobin, total bilirubin, NLR, and PLR. Model 3 (surgery-related confounders) included: blood loss, colloid, crystalloid, NSAIDs, blood transfusion, morphine, total remifentanil dosage, glucose, and dexmedetomidine. Model 4 (fully adjusted) included all of the above confounders. Results from univariate analysis for all covariates in Model 4 are presented in Supplementary Table S3, and univariate results in the PSM cohort are provided in Supplementary Table S4.

To reduce confounding, 1:1 propensity score matching (PSM) was performed using logistic regression, incorporating all covariates described above. Covariate balance was assessed using standardized mean difference (SMD), with SMD < 0.2 considered acceptable (20). Logistic regression was then repeated in the matched cohort to confirm associations.

Results were reported as odds ratios (ORs) with 95% confidence intervals (CIs). A *p* < 0.05 was considered statistically significant. All analyses were performed using R software (version 4.0.3, R Foundation for Statistical Computing, Vienna, Austria) with relevant packages including tableone, MatchIt, pROC, Matching, Cobalt, and rms.

## Results

### Epidemiology and overall survival

Among 178 patients with perioperative ischemic stroke, the overall incidence of postoperative delirium (POD) was 40.4% (72/178), underscoring the substantial burden in this high-risk population. During follow-up, 87 patients (52.7%) died.

**Figure 2:**
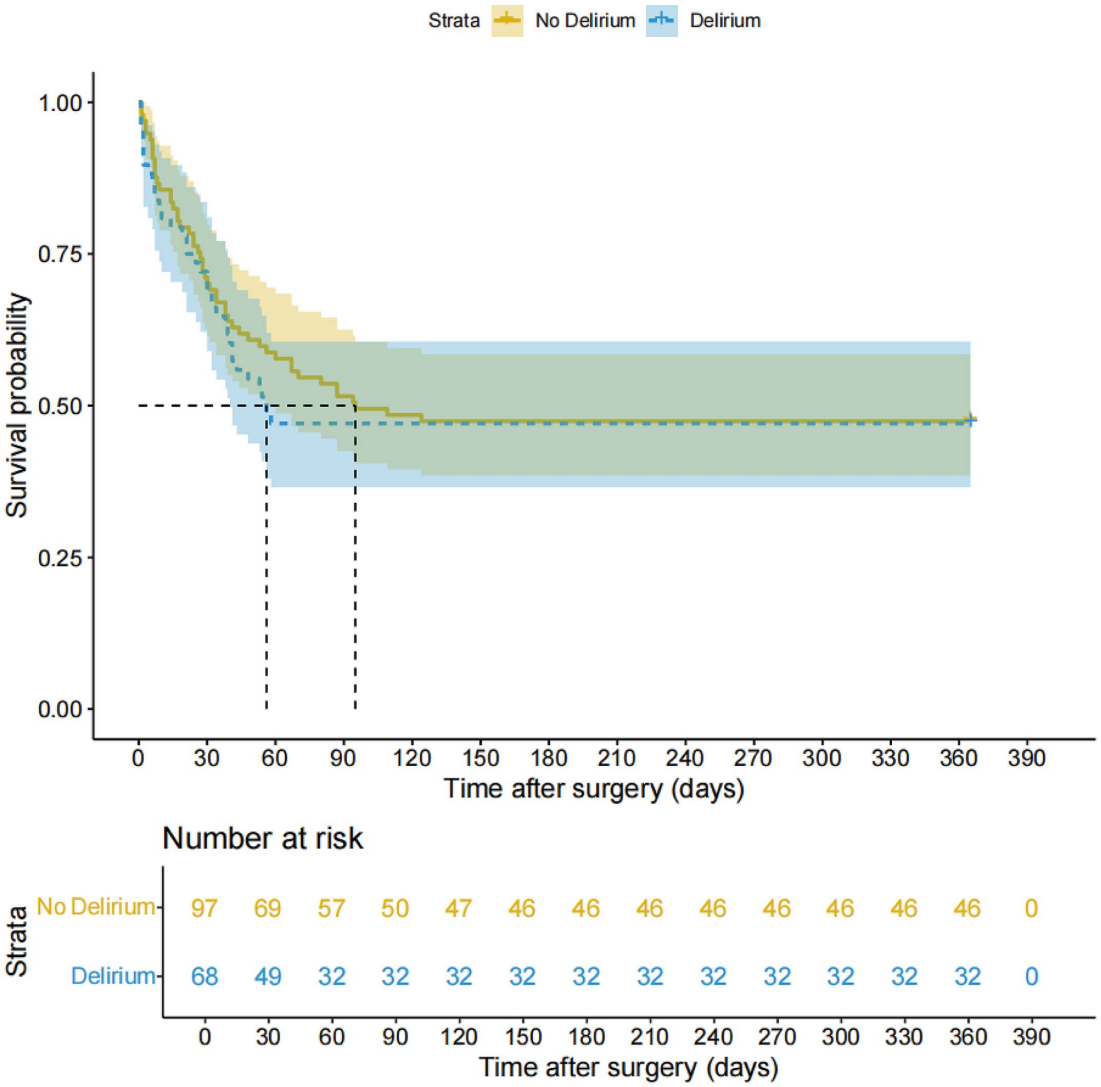
Kaplan–Meier survival curves for perioperative ischemic stroke patients stratified by postoperative delirium status. The curves compare overall survival between patients with POD (*n* = 72) and without POD (*n* = 106). Median survival was 67 days (IQR: 18–75) in the POD group versus 365 days (IQR: 30–365) in the non-POD group. Log-rank test: *p* = 0.156; Cox regression hazard ratio (HR): 1.068 (95% CI: 0.697–1.637), *p* = 0.762.

Kaplan–Meier survival analysis showed no statistically significant difference in overall survival between patients with and without POD (*p* = 0.156, Figure 2). Median survival was 365 days (IQR: 30–365) for patients without delirium and 67 days (IQR: 18–75) for those with delirium. Although delirium was associated with a trend toward shorter survival, this did not reach statistical significance. Cox regression analysis confirmed that POD was not significantly associated with overall survival (HR: 1.068; 95% CI: 0.697–1.637; *p* = 0.762; Supplementary Table S2). Baseline characteristics of the study population are presented in Table 1.

**Table 1:** Baseline characteristics unadjusted sample and propensity score-matched sample.

Characteristic	Unadjusted sample (*N* = 178)			PSM adjusted (1:1) (*N* = 88)		
	Fentanyl (*n* = 73)	Sufentanil (*n* = 105)	SMD	Fentanyl (*n* = 44)	Sufentanil (*n* = 44)	SMD
Delirium (%)			55.661			0.978
No	68 (93.2)	38 (36.2)		39 (88.6)	21 (47.7)	
Yes	5 (6.8)	67 (63.8)		5 (11.4)	23 (52.3)	
Sex (%)			0.071			0.137
Female	35 (47.9)	47 (44.8)		25 (56.8)	22 (50.0)	
Male	38 (52.1)	58 (55.2)		19 (43.2)	22 (50.0)	
Age (median [IQR])	73 (70,75)	71 (67,76)	3.482	72.00 [69.00, 75.00]	71.50 [68.75, 77.00]	0.053
BMI (median [IQR])	24.39 [22.65,26.89]	24.22 [22.49,26.04]	0.262	24.97 [22.61, 26.84]	23.52 [21.63, 25.97]	0.196
ASA (%)			3.799			0.103
Class I	2 (2.7)	0		0 (0.0)	0 (0.0)	
Class II	45 (61.6)	70 (66.7)		31 (70.5)	29 (65.9)	
Class III	22 (30.1)	32 (30.5)		11 (25.0)	13 (29.5)	
Class IV	4 (5.5)	3 (2.9)		2 (4.5)	2 (4.5)	
Hypertension (%)			9.733			0.138
No	46 (63)	40 (38.1)		23 (52.3)	26 (59.1)	
Yes	27 (37)	65 (61.9)		21 (47.7)	18 (40.9)	
Coronary heart disease (%)			2.502			0.069
No	64 (87.7)	81 (77.1)		38 (86.4)	39 (88.6)	
Yes	9 (12.3)	24 (22.9)		6 (13.6)	5 (11.4)	
Atrial fibrillation (%)			0.231			0.125
No	72 (98.6)	99 (94.3)		43 (97.7)	42 (95.5)	
Yes	1 (1.4)	6 (5.7)		1 (2.3)	2 (4.5)	
Previous ischemic stroke (%)			0			0.143
No	45 (61.6)	65 (61.9)		30 (68.2)	27 (61.4)	
Yes	28 (38.4)	40 (38.1)		14 (31.8)	17 (38.6)	
Diabetes (%)			0.999			0.048
No	50 (68.5)	63 (60)		29 (65.9)	28 (63.6)	
Yes	23 (31.5)	42 (40)		15 (34.1)	16 (36.4)	
Peripheral vascular diseases (%)			0.771			0.069
No	66 (90.4)	89 (84.8)		38 (86.4)	39 (88.6)	
Yes	7 (9.6)	16 (15.2)		6 (13.6)	5 (11.4)	
Renal dysfunction (%)			0.958			0.216
No	71 (97.3)	102 (97.1)		43 (97.7)	44 (100.0)	
Yes	2 (2.7)	3 (2.9)		1 (2.3)	0 (0.0)	
Hb (median [IQR])	128 (113,143)	129 (121,140)	0.022	128.50 [120.00, 143.50]	131.50 [121.00, 140.25]	0.026
Total Bilirubin (median [IQR])	11.2 (7.7,16.6)	11 (8.2,14.5)	0.11	9.15 [7.22, 13.55]	11.40 [8.67, 14.62]	0.138
Glu (median [IQR])	5.36 (4.74,6.28)	5.7 (4.85,7.42)	2.411	5.62 [4.80, 6.45]	5.04 [4.80, 6.72]	0.023
NSAIDs (%)			6.363			0.173
No	19 (26)	11 (10.5)		10 (22.7)	7 (15.9)	
Yes	54 (74)	94 (89.5)		34 (77.3)	37 (84.1)	
Blood Loss (median [IQR])	200.00 [50.00,300.00]	150.00 [50.00,300.00]	0.203	200.00 [87.50, 300.00]	150.00 [100.00, 212.50]	0.032
Colloid (median [IQR])	500.00 [500.00,1000.00]	500.00 [500.00,500.00]	3.078	500.00 [500.00, 1000.00]	500.00 [500.00, 500.00]	0.058
Crystalloid (median [IQR])	1350.00[1000.00,1750.00]	1600.00[1100.00,2150.00]	4.583	1500.00 [1100.00, 1987.50]	1200.00 [1000.00, 1725.00]	0.177
Dexmedetomidine (%)			37.836			0.22
No	25 (34.2)	85 (81)		43 (97.7)	41 (93.2)	
Yes	48 (65.8)	20 (19)		1 (2.3)	3 (6.8)	
NLR (median [IQR])	2.37 [1.73,3.84]	2.21 [1.65,3.29]	0.756	2.17 [1.70, 3.81]	1.97 [1.49, 3.28]	0.076
PLR (median [IQR])	131.55 [94.74,205.40]	135.06 [100.20,170.25]	0.055	130.98 [90.11, 193.67]	135.74 [90.05, 184.65]	0.06
Blood transfusion (%)			0.078			0.06
No	57 (78.1)	85 (81)		37 (84.1)	36 (81.8)	
Yes	16 (21.9)	20 (19)		7 (15.9)	8 (18.2)	
Morphine (median [IQR])	120.00 [90.00,150.00]	150.00 [105.00,165.00]	7.664	120.00 [90.00, 150.00]	120.00 [75.00, 150.00]	0.075
Total remifentanil dosage (median [IQR])	1752.00 [1053.33,2560.00]	1710.00 [1053.33,2453.33]	0.035	1718.67 [1088.33, 2453.33]	1621.33 [958.33, 2355.83]	0.084
Hospital stay duration	13.00 [8.00, 18.00]	12.00 [8.00, 18.00]	0.11	11.00 [7.00, 16.00]	12.00 [8.00, 18.25]	0.353

The data are shown as the median (interquartile range), *n* (%), or mean ± SD.

PSM, propensity score matching; SMD, standardized mean difference; ASA, American Society of Anesthesiologists; Hb, hemoglobin; Glu, glucose; NSAIDs, non-steroidal anti-inflammatory drugs; NLR, neutrophil-to-lymphocyte ratio; PLR, platelet-to-lymphocyte ratio.

### Primary analysis

Seventy-two patients developed POD. In unadjusted logistic regression, fentanyl use was significantly associated with a lower risk of POD compared to sufentanil (OR: 0.042; 95% CI: 0.014–0.103; *p* = 0.005; Table 2). This association of lower POD risk with fentanyl compared to sufentanil persisted after adjusting for patient-related factors (OR: 0.025; 95% CI: 0.006–0.079; *p* < 0.001), surgery-related confounders (OR: 0.039; 95% CI: 0.011–0.112; *p* < 0.001), and all predefined confounders (OR: 0.017; 95% CI: 0.003–0.070; *p* = 0.017; Table 2).

**Table 2:** Logistic regression and propensity score analysis of the association between fentanyl and postoperative delirium.

Analysis method	OR	95% CI	*p* value
Logistic regression analysis (*N* = 178)			
Model 1 (unadjusted)^*^	0.042	0.014–0.103	<0.001
Model 2 (patient-related confounders adjusted)^†^	0.025	0.006–0.079	<0.001
Model 3 (surgery-related confounders adjusted)^‡^	0.039	0.011–0.112	<0.001
Model 4 (fully adjusted)^§^	0.017	0.003–0.070	<0.001
Propensity score analysis			
PS matching (*N* = 88)^#^	0.005	0–0.057	<0.001

CI, confidence interval; OR, odds ratio; PS, propensity score.

* Model 1 was a univariable crude model.

† Model 2 included sex, Age, BMI, ASA, Hypertension, Diabetes, Coronary heart disease, Previous ischemic stroke, Arterialfibrillation, Peripheral vascular diseases, Renal dysfunction, Hemoglobin, Total Bilirubin, NLR, PLR.

‡ Model 3 included Blood Loss, Colloid, Crystalloid, NSAIDs, Blood transfusion, Morphine, Total remifentanil dosage, Glu, Dexmedetomidine.

§ Model 4 includes all the above confounders. Full results are displayed in Supplementary Table S3.

# 88 Pairs were matched by propensity score. Full results are displayed in Supplementary Table S4.

After propensity score matching (PSM), fentanyl remained associated with significantly lower POD risk compared to sufentanil (OR:0.005; 95% CI: 0–0.057; *p* < 0.001). Detailed results are provided in Supplementary Table S3.

### PSM analysis

**Figure 3:**
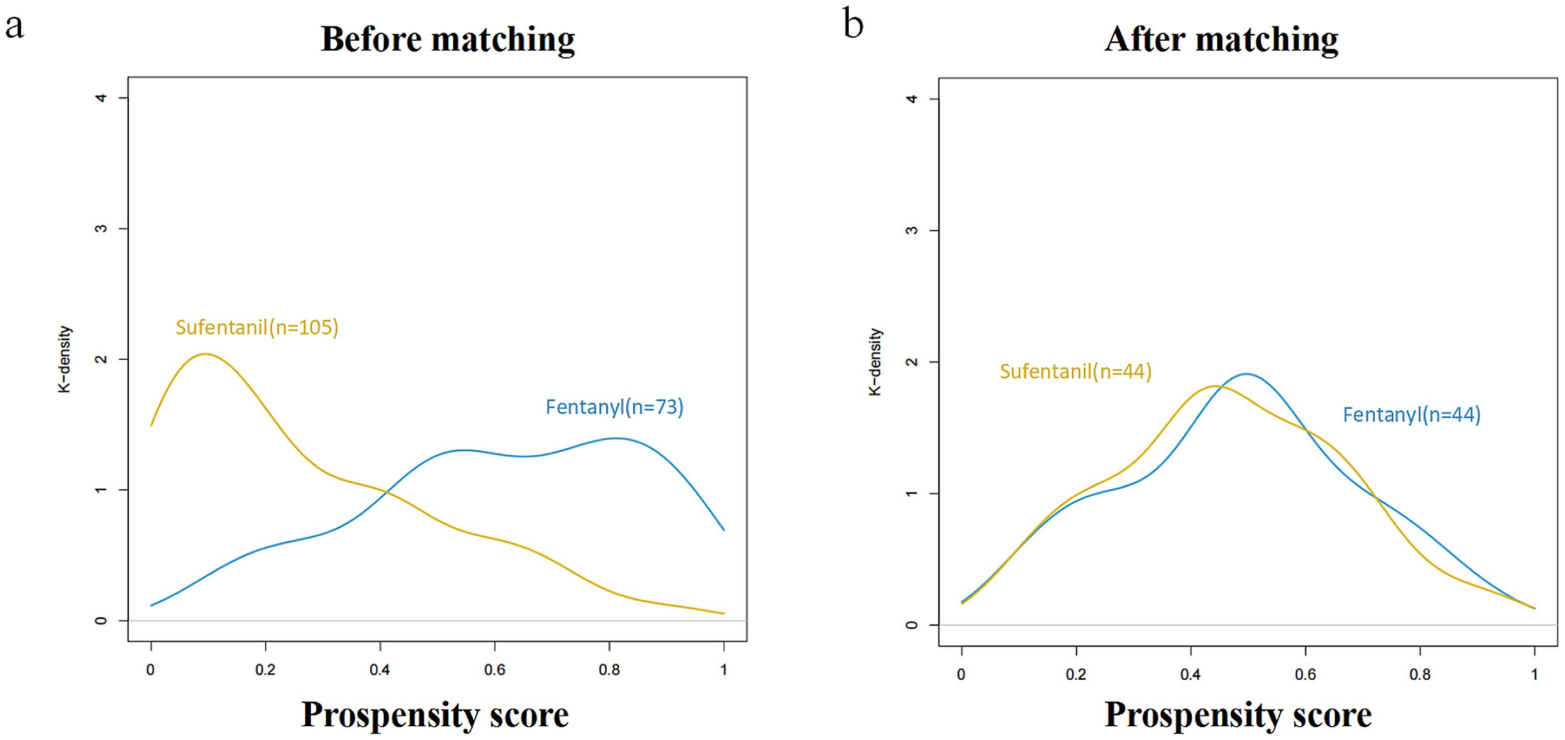
Propensity score histograms for the two groups. **(a)** Before matching: fentanyl group (*n* = 73) and sufentanil group (*n* = 105). **(b)** After matching: fentanyl group (*n* = 44) and sufentanil group (*n* = 44). Standardized mean differences (SMD) for most covariates were < 0.2 after matching, indicating adequate balance between groups.

PSM generated matched cohorts of 44 patients each in the fentanyl and sufentanil groups, with standardized mean differences < 0.2 for most covariates (Figures 3a,b). Logistic regression in the matched cohort confirmed a significantly lower POD risk with fentanyl (OR: 0.005; 95% CI: 0–0.057; *p* < 0.001; Table 2). The association was consistent across both unadjusted and adjusted analyses, reinforcing the robustness of the findings. Additional PSM details are presented in Supplementary Table S4.

### Subgroup analysis

Subgroup analyses by sex, age (≤70 *vs*. >70 years), and diabetes status showed that fentanyl consistently reduced POD risk across all categories: females (OR: 0.001, 95% CI: 0–0.061, *p* = 0.00934), males (OR: 0, 95% CI: 0–0.003, *p* = 0.00146), age ≤70 years (OR: 0.006, 95% CI: 0–0.177, *p* = 0.0168), age >70 years (OR: 0.012, 95% CI: 0.001–0.088, *p* < 0.001), non-diabetic patients (OR: 0.001, 95% CI: 0–0.017, *p* < 0.001), and diabetic patients (OR: 0.007, 95% CI: 0–0.124, *p* = 0.0104).

**Figure 4:**
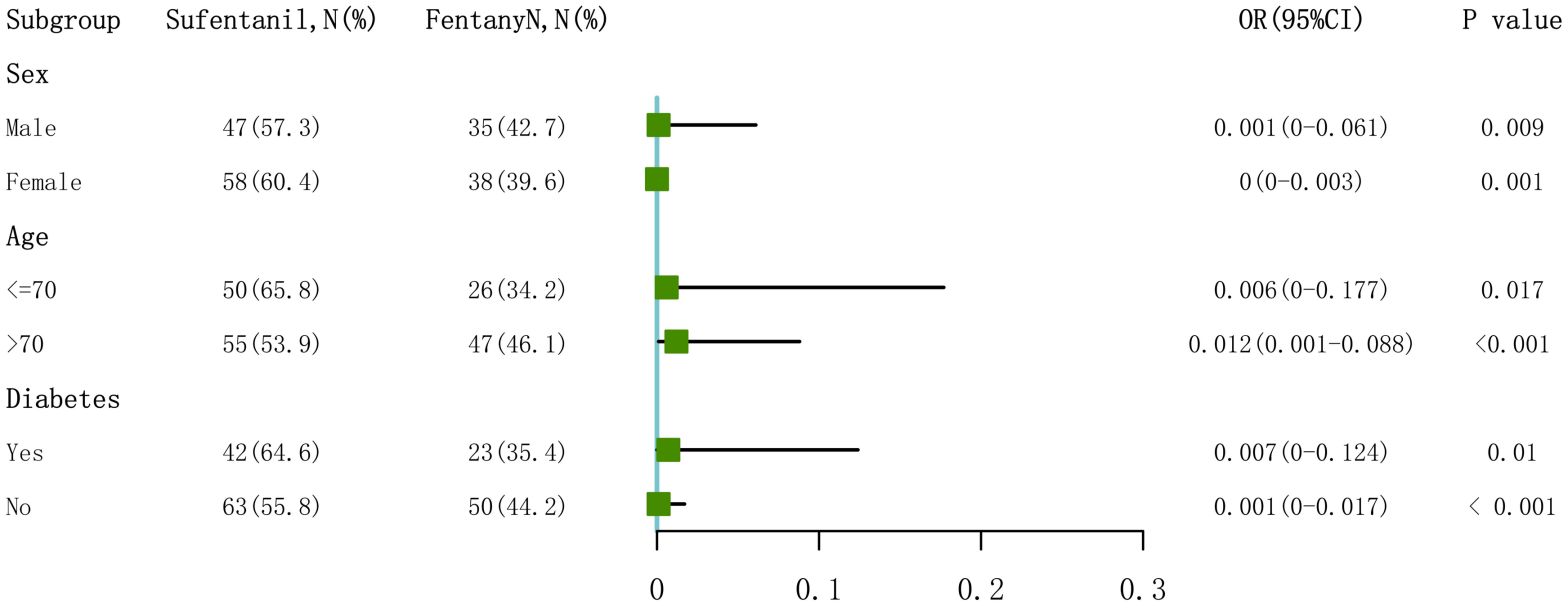
Subgroup analysis of the association between fentanyl use and postoperative delirium risk. Forest plot showing odds ratios (OR) with 95% confidence intervals for the association between fentanyl versus sufentanil and postoperative delirium risk across pre-specified subgroups (*n* = 178 total). No significant interaction was observed (all *p*-interaction > 0.05), indicating consistent lower POD risk with fentanyl compared to sufentanil across all patient subgroups. OR, odds ratio.

No significant interaction was observed between any subgroup variable and the lower POD risk associated with fentanyl versus sufentanil (*p*_-interaction_ > 0.05), indicating that the lower POD risk with fentanyl compared to sufentanil was consistent across patient subgroups (Figure 4).

## Discussion

This study builds on our long-standing perioperative stroke research program, which has established one of China’s largest multi-center databases comprising over 200,000 non-cardiac surgical cases (5–11). Through this program, we have identified multiple risk factors, developed validated prediction models, and investigated related complications, including postoperative delirium (21). The present work extends that framework by examining whether intraoperative opioid choice influences delirium risk in the highest-risk group.

We found a 40.4% incidence of delirium in this population, highlighting the substantial burden when surgical stress is layered onto existing cerebrovascular injury. This rate closely mirrors those reported in community-acquired stroke populations, where post-stroke delirium occurs in 13–48% of patients depending on stroke severity and baseline characteristics (4). Shi et al. reported a pooled delirium prevalence of 23% in acute stroke patients, with higher rates in severe strokes and elderly populations (22). Our findings suggest that perioperative stroke patients face similar delirium risks to community stroke patients, reinforcing the persistent vulnerability of stroke patients to cognitive complications regardless of setting.

Survival analysis revealed a concerning prognosis: patients with delirium had a median survival of just 67 days compared to the 365 days in those without delirium, and overall mortality was 52.7%. While the difference in survival was not statistically significant (*p* = 0.156, HR: 1.068, 95% CI: 0.697–1.637), the numerical gap underscores the clinical importance of preventing delirium in this population, particularly by targeting modifiable risk factors. These findings align with recent meta-analyses demonstrating significantly worse prognosis for stroke patients with delirium, including a 4.71-fold increased risk of in-hospital mortality (95% CI: 1.85–11.96), a 4.91-fold increased risk of 12-month mortality (95% CI: 3.18–7.6), and a 3.3-fold increased risk of 5-year mortality (23).

Previous research has linked advanced age, preexisting cognitive impairment, polypharmacy, major surgery, and specific anesthetic agents with increased delirium risk (24). While the effects of anesthetic techniques on postoperative cognition in high-risk patients have been studied (25), the impact of specific opioid choice in perioperative ischemic stroke has been largely overlooked.

Opioid selection during anesthesia is one such factor. Fentanyl and sufentanil, though both synthetic opioids, differ in pharmacokinetics and pharmacodynamics, which may influence neurological outcomes (26). Recent studies indicate that opioids can affect neuroinflammation and blood–brain barrier integrity, which are key processes in both stroke and delirium pathophysiology (27). In our analysis, fentanyl was associated with a markedly lower delirium risk compared to sufentanil (OR: 0.017 in fully adjusted models), and this association held across all analytical approaches, including propensity score matching, suggesting a true difference in delirium risk between the two opioids rather than a statistical artifact. To our knowledge, this represents the first evidence comparing fentanyl and sufentanil for delirium risk in perioperative stroke patients. Several mechanisms could explain fentanyl’s apparent advantage. Its rapid onset and shorter context-sensitive half-time compared to sufentanil may reduce residual CNS depression in the early postoperative period (26). Differences in how these opioids affect cerebral blood flow autoregulation may also play a role, especially in stroke patients with impaired cerebrovascular reserve (16). Furthermore, experimental evidence suggests variable opioid effects on neuroinflammatory signaling and blood–brain barrier stability (27). Specifically, sufentanil’s higher lipophilicity and greater mu-opioid receptor affinity may lead to prolonged central nervous system effects and enhanced microglial activation, promoting neuroinflammatory cascades that predispose to delirium (28). In contrast, fentanyl’s faster redistribution may limit sustained receptor occupancy and attenuate inflammatory cytokine release (29). Moreover, in patients with compromised cerebral autoregulation following ischemic stroke, even subtle differences in opioid-induced hemodynamic effects could differentially impact cerebral perfusion pressure and blood–brain barrier permeability, thereby influencing delirium susceptibility. The consistently lower delirium risk with fentanyl versus sufentanil across all subgroups, including older adults (OR: 0.012 in those >70 years), supports its potential as the preferred opioid in this context.

The clinical implications are immediate: opioid selection lies entirely within the anesthesiologist’s control, representing a straightforward strategy to potentially reduce delirium burden. This aligns with broader perioperative brain health initiatives advocating for anesthetic optimization in high-risk populations, specifically the perspective that anesthesiologists should systematically tailor anesthetic choices to protect neurological outcomes in vulnerable patient populations (30). The consistency of the effect across subgroups and the large magnitude of risk reduction (>95% in matched analysis) underscore its potential impact.

In summary, this large-scale database study demonstrates that fentanyl’s association with lower POD risk in perioperative stroke patients suggests opioid pharmacology may influence delirium susceptibility through mechanisms beyond analgesia alone, warranting further mechanistic investigation.

### Study strengths and limitations

This study has several notable strengths. It specifically targeted perioperative ischemic stroke patients—a group particularly high-risk for delirium—using a large, detailed dataset that included comprehensive patient characteristics and anesthetic management variables. This granularity enabled a thorough analysis of delirium risk factors. The observed association of lower delirium risk with fentanyl compared to sufentanil was confirmed through multiple statistical approaches, including propensity score matching, which enhances the robustness of the findings. Importantly, the study centered on opioid choice, an aspect within the anesthesiologist’s direct control.

However, there are important limitations. Delirium is a multifactorial condition, making prediction based on opioid selection alone inherently challenging. The retrospective, single-center design limits the generalizability of our findings to other healthcare settings with different patient populations, surgical practices, or anesthetic protocols. Our results may remain susceptible to unmeasured confounding despite extensive adjustment. Key factors such as anesthesia depth, postoperative pain control strategies, and detailed stroke characteristics were not fully captured but may have influenced outcomes. Moreover, while propensity score matching was employed to minimize confounding, the specific types of surgical procedures were not included as a covariate in our models; given that delirium incidence varies substantially with surgical stress and tissue trauma extent, this represents a potential source of residual confounding. Similarly, detailed information regarding anesthesia type (regional, general, combined, or multimodal approaches) was not fully transparent in the study population, and more granular inclusion and exclusion criteria related to these factors would strengthen future investigations. Critically, we did not assess long-term cognitive outcomes beyond the acute postoperative period, precluding conclusions about persistent cognitive impairment or functional recovery trajectories. Additionally, although dexmedetomidine and NSAIDs were included as covariates in our models, residual confounding from these agents cannot be entirely excluded, as their dosing, timing, and indications may have varied and potentially influenced delirium outcomes independently. Beyond these factors, other potential sources of bias warrant consideration: selection bias may have arisen from the retrospective identification of perioperative stroke cases, as milder or undiagnosed strokes could have been missed; information bias related to delirium ascertainment may exist given the reliance on clinical documentation rather than standardized prospective screening; temporal changes in clinical practice over the 11-year study period (2008–2019) could introduce era effects; indication bias cannot be excluded as opioid selection may have been influenced by unmeasured patient characteristics or physician preferences correlated with delirium risk; and survivor bias may affect our findings if patients who died early had different opioid exposures. Future multi-center prospective trials are needed to validate these results and to define optimal opioid protocols for perioperative ischemic stroke patients.

## Conclusion

In this large-scale database study, intraoperative fentanyl use was associated with a significantly lower risk of postoperative delirium compared to sufentanil in perioperative ischemic stroke patients. Opioid selection represents a readily modifiable aspect of anesthetic care that may substantially influence delirium risk and provide actionable evidence to inform anesthetic management in this vulnerable population.

## Data Availability

The original contributions presented in the study are included in the article/Supplementary material, further inquiries can be directed to the corresponding authors.
