# Supplementary material for: Sufentanil increases delirium risk compared to fentanyl in elderly perioperative ischemic stroke patients: a retrospective cohort study
Source: Front Med (Lausanne). 2026 Feb 12;13:1770366. doi: 10.3389/fmed.2026.1770366 (PMC12936512; doi:10.3389/fmed.2026.1770366)
Supplement: Supplementary file 1 [file Supplementary_file_1.docx]

SUPPLEMENTAL MATERIAL

**Supplementary Table S1.** ICD-9/10 Diagnosis Codes for Ischemic Stroke

| Ischemic stroke | ICD-9/ICD-10 | 433.X1/I63.X | Occlusion and stenosis of precerebral arteries with cerebral infarction |
| --- | --- | --- | --- |
|  | ICD-9 | 434.X1 | Occlusion of cerebral arteries with cerebral infarction |
|  | ICD-9/ICD-10 | 437.1/I67.81,  I67.89 | Other generalized ischemic cerebrovascular disease |
|  | ICD-9/ICD-10 | 437.9/I67.9 | Unspecified cerebrovascular disease |

**Supplementary Table S2**. Cox regression analysis of the association between postoperative delirium and overall survival in perioperative ischemic stroke patients.

| **Variables** | **Cox regression analysis** | | |
| --- | --- | --- | --- |
|  | **HR** | **CI** | **P** |
| **Delirium** | 1.06827260101846 | 0.697-1.637 | 0.762 |

| Abbreviations: HR, hazard ratio; CI, confidence interval. |
| --- |

**Supplementary Table S3**. Univariate and Multivariate Logistic Regression Analysis for Postoperative delirium in Model 4.

| **Variables** | **Logistic regression analysis** | | |
| --- | --- | --- | --- |
|  | **OR** | **CI** | **P** |
| **Fentanyl** | 0.017 | 0.003-0.07 | <0.001 |
| **Sex** | 0.679 | 0.227-1.954 | 0.057 |
| **Age** | 1.213 | 1.08-1.383 | 0.003 |
| **BMI** | 1.072 | 0.908-1.261 | 0.781 |
| **ASA physical status** |  |  |  |
| Class II | 400375.241889211 | 0-NA | 0.738 |
| Class III | 281729.935732743 | 0-NA | 0.174 |
| Class Ⅳ | 4017065.43563308 | 0-NA | 0.279 |
| **Hypertension** | 2.367 | 0.824-7.048 | 0.545 |
| **Diabetes** | 2.051 | 0.644-6.957 | 0.774 |
| **Coronary heart disease** | 0.969 | 0.26-3.592 | 0.786 |
| **Previous ischemic stroke** | 0.328 | 0.104-0.94 | 0.241 |
| **Atrial fibrillation** | 0.322 | 0.025-4.645 | 0.473 |
| **Peripheral vascular diseases** | 0.835 | 0.168-4.206 | 0.996 |
| **Renal dysfunction** | 20.829 | 0.772-907.419 | 0.200 |
| **Hb** | 1.002 | 0.972-1.034 | 0.068 |
| **Total Bilirubin** | 1.007 | 0.993-1.023 | 0.864 |
| **NLR** | 0.991 | 0.869-1.117 | 0.132 |
| **PLR** | 1.002 | 0.996-1.008 | 0.504 |
| **Blood Loss** | 1.001 | 0.999-1.003 | 0.878 |
| **Colloid** | 1.000 | 0.998-1.001 | 0.587 |
| **Crystalloid** | 1.000 | 0.999-1.001 | 0.536 |
| **NSAIDs** | 0.448 | 0.092-1.955 | 0.135 |
| **Blood transfusion** | 0.348 | 0.063-1.647 | 0.040 |
| **Morphine** | 1.033 | 1.017-1.052 | 0.292 |
| **Total Remifentanil Dosage** | 1.000 | 0.999-1 | 0.824 |
| **Glu** | 1.088 | 0.868-1.389 | 0.365 |
| **Dexmedetomidine** | 0.957 | 0.203-4.448 | <0.001 |
| **Hospital Stay Duration** | 4.25686208727768 | 0.085-212.995 | 0.465981820255696 |
| \| Abbreviations: BMI, body mass index; ASA, American Society of Anesthesiologists; Hb, hemoglobin; Glu, Glucose; NSAIDs, non-steroidal anti-inflammatory Drugs; NLR, neutrophil-to-lymphocyte Ratio; PLR, platelet-to-lymphocyte ratio. \| \| --- \| | | | |

**Supplementary Table S4**. Univariate Logistic Regression Analysis for Postoperative delirium in the PSM Cohort.

| **Variables** | **Logistic regression analysis** | | |
| --- | --- | --- | --- |
|  | **OR** | **CI** | **P** |
| **Fentanyl** | 0.005 | 0-0.057 | <0.001 |
| **Sex** | 0.123 | 0.011-0.975 | 0.057 |
| **Age** | 1.496 | 1.183-2.067 | 0.003 |
| **BMI** | 1.052 | 0.724-1.476 | 0.781 |
| **ASA physical status** |  |  |  |
| Class III | 1.647 | 0.075-29.273 | 0.738 |
| Class Ⅳ | 193.423 | 0.32-13336265.87 | 0.174 |
| **Hypertension** | 2.867 | 0.451-23.004 | 0.279 |
| **Diabetes** | 1.995 | 0.205-21.576 | 0.545 |
| **Coronary heart disease** | 0.609 | 0.018-18.936 | 0.774 |
| **Previous ischemic stroke** | 0.752 | 0.091-6.099 | 0.786 |
| **Arterial fibrillation** | 0.015 | 0-73.374 | 0.241 |
| **Peripheral vascular diseases** | 3.212 | 0.124-93.542 | 0.473 |
| **Renal dysfunction** | 0.000 | NA-3.42875495928471e+62 | 0.996 |
| **Hb** | 1.047 | 0.982-1.135 | 0.200 |
| **Total Bilirubin** | 1.072 | 1.003-1.158 | 0.068 |
| **NLR** | 0.963 | 0.53-1.408 | 0.864 |
| **PLR** | 1.013 | 0.996-1.032 | 0.132 |
| **Blood Loss** | 1.002 | 0.996-1.009 | 0.504 |
| **Colloid** | 1.000 | 0.996-1.004 | 0.878 |
| **Crystalloid** | 1.001 | 0.999-1.003 | 0.587 |
| **NSAIDs** | 0.504 | 0.05-4.401 | 0.536 |
| **Blood transfusion** | 0.057 | 0.001-2.14 | 0.135 |
| **Morphine** | 1.054 | 1.011-1.12 | 0.040 |
| **Total Remifentanil Dosage** | 0.999 | 0.997-1.001 | 0.292 |
| **Glu** | 0.933 | 0.501-1.742 | 0.824 |
| **Dexmedetomidine** | 0.183 | 0.003-6.038 | 0.365 |
| **Hospital Stay Duration** | 68.530076868431 | 0.43-10931.53 | 0.101202897 |

| Abbreviations: BMI, body mass index; ASA, American Society of Anesthesiologists; Hb, hemoglobin; Glu, Glucose; NSAIDs, non-steroidal anti-inflammatory Drugs; NLR, neutrophil-to-lymphocyte Ratio; PLR, platelet-to-lymphocyte ratio. |
| --- |
